# The Master Clinician’s Elective: Integrating Evidence-Based Physical Examination and Point of Care Ultrasonography in Modern Clinical Medicine

**DOI:** 10.24908/pocus.v5i1.14225

**Published:** 2020-07-06

**Authors:** Maria Gabriela Frank, Cason Pierce, Noelle Northcutt, Joseph Walker Keach, Gerard Salame, Rebecca Allyn

**Affiliations:** 1 Division of Hospital Medicine, Department of Medicine, Denver Health Hospital Authority Denver, CO; 2 University of Colorado School of Medicine Aurora, CO

**Keywords:** POCUS, education

## Abstract

**Background:** Many internal medicine residency programs have incorporated ultrasonography into their curriculum; however, its integration with physical examination skills teaching at a graduate medical level is scarce. The program’s aim is to create a reproducible elective that combines physical exam and bedside ultrasound as a method for augmenting residents’ knowledge and competence in these techniques with the ultimate goal of improving patient care. **Methods**: We designed and implemented a two-week elective rotation for senior internal medicine residents, combining evidence-based physical examination with diagnostic bedside ultrasonography. The rotation took place in an inpatient setting at Denver Health Hospital. Program evaluation data was collected data between February 2016 to March 2019. IRB approval was waived. **Results:** Since its inception in 2016, 19 residents completed the rotation. Residents performed a pre-test and a post-test under direct observation by course faculty. Each resident was measured on the ability to perform pre-determined physical exam and point-of-care ultrasound (POCUS) skills. In the pre-test, participants correctly performed an average of 40% of expected physical exam maneuvers and 32% of expected POCUS skills. At elective conclusion, all participants were effectively able to demonstrate the highest yield physical exam and ultrasound maneuvers. **Discussion and Conclusion:** An elective designed specifically to integrate POCUS and physical exam modalities improves the ability of resident physicians to utilize both diagnostic modalities.

## Background

Medical schools and training programs in the United States have shifted emphasis away from physical exam teaching 1, 2. A recent study identified that students perform worse on the physical examination components of the United States Medical License Examination Step 2 Clinical Skills relative to the history taking components 3. While modern medical imaging positively impacts patient care in many ways, its widespread availability has decreased practitioners’ reliance on the physical exam for establishing diagnoses, reduced the confidence of trainees and practitioners in their physical examination skills, and eroded their perceived value of the physical examination 4, 5. In turn, faculty feel unqualified and less motivated to teach these skills, further perpetuating their deterioration 4, 5.

A review by Oliver et. al. 1revealed a decline of 31.2% from 1975 to 2011 in the number of total body systems documented as examined by house-staff and junior faculty alike. In its Choosing Wisely® campaign, the American Board of Internal Medicine highlights the downstream effects of blind reliance on technological innovation in the practice of medicine: increased healthcare expenditures, medically unnecessary interventions, and adverse patient outcomes 6, 7. Within this context, our elective provides learners with a more tempered integration of a new imaging modality—Point of Care Ultrasonography (POCUS)—into clinical practice with specific goals: to answer a focused clinical question, improve procedural safety, minimize complications, and augment the accuracy of the physical examination.

Additional motivators for our elective include: 1) the expansion of POCUS into inpatient medicine; 2) a national trend to incorporate ultrasound Internal Medicine residency training programs 8; 3) a growing body of literature supporting the value of POCUS in improving accuracy of the physical exam 9, 10, 11, 12, 13, 14, 15; and 4) technological improvements in POCUS technology that make it accessible and affordable for individual practitioners. 

Acknowledging the persistent value of the physical exam while recognizing the need to teach ultrasound skills to future physicians, we sought to synthesize the subjects into a two-week “Master Clinician” elective. 

## Methods

We designed and implemented a two-week elective, offered yearly since 2016, combining teaching of EBPE skills with POCUS. In order to perform the program evaluation, we collected and analyzed data between February 2016 and March 2019. Six faculty members from the division of Hospital Medicine at Denver Health Hospital Authority (DHHA), an Internal Medicine Residency Program (IMRP) affiliated site for the University of Colorado, School of Medicine (CUSOM) facilitated the curriculum and taught the content at DHHA. The elective was offered for up to six PGY-2 and PGY-3 categorical Internal Medicine residents in the CUSOM IMRP per course iteration. Since inception, 19 residents have participated. On average, 20 were on the waitlist each year.

### Program’s Development

The program’s main objective was to create a reproducible and effective elective rotation combining evidence-based physical exam (EBPE) and POCUS as a method for cultivating resident’s knowledge and competence in these techniques, ultimately leading to improved quality of care and patient safety. 

The program’s aims included: 1) use of case-based-learning to identify gaps in residents’ skills; 2) employ direct observation to provide learners real-time, targeted feedback; 3) analyze published literature on discussed topics; 4) correlate ultrasound with physical examination findings; 5) provide guidance on how to integrate this information into clinical practice 6) test newly acquired knowledge and skills through near-peer teaching.

By the end of the elective rotation, participants were able to: 1) understand the utility, importance, and evidence behind common and uncommon physical exam maneuvers; 2) demonstrate the ability to apply likelihood ratios (LR) to modify diagnostic probability; 3) exhibit the aptitude to implement an EBPE by correctly performing high-yield physical exam maneuvers, accurately identifying the presence of pathology during examination, and synthesizing signs with clinical decision making; 4) confirm or challenge clinical diagnosis by skillfully performing high yield POCUS maneuvers; 5) utilize POCUS to increase success rates and decrease complications of medical bedside procedures. Institutional Review Board approval was not required for this study.

### Program’s Implementation

To ensure integration, each instructional day provided EBPE teaching and POCUS training sessions focused on a single content area. We organized each day as follows: one hour of independent reading and literature review, followed by one-hour long interactive EBPE didactic. This was followed by two hours of EBPE bedside-rounding led by faculty on prescreened patients with pathology relating to that day’s topic. In the afternoon, interactive pedagogies continued with a one-hour POCUS didactic session, followed by two and a half hours of POCUS rounding, led and supervised a trained faculty. 

Session topics (Table 1) and clinical cases were selected based on the relevance to hospital medicine (commonly encountered diagnosis) and on published literature in EBPE and POCUS (Table 2). During EBPE didactics faculty highlighted the limited diagnostic value of commonly performed exam maneuvers, subsequently discussing and demonstrating specific exam maneuvers with the highest diagnostic yield. Course participants practiced these maneuvers under direct observation of faculty while receiving feedback. POCUS content focused on: image acquisition, identification of pathologic findings, and integration into clinical reasoning. Image acquisition was taught systematically for each application and can be deconstructed as follows: 1) visualization during a short didactic lecture; 2) hands-on guided scanning with one of the two POCUS content faculty; 3) practicing during unguided free-scanning; 4) quality assessment guided by POCUS faculty. Integration of POCUS into clinical reasoning aimed to supplement the EBPE using systematic approaches to scanning in each content area and body region. Course participants then demonstrated POCUS knowledge acquisition at course end by preparing and presenting key concepts to their peers during a noon conference, with POCUS faculty on-hand to guide discussion and ensure accuracy. Enrollment was capped to optimize the participant to attending ratio, which was maintained at a 5:1 ratio.

**Table 1:** Topic pairs for BPE and POCUS didactics

**Evidence-Based Physical Exam Sessions (and some examples of didactic descriptions)**	**Topics for Point-of-Care Ultrasonography Didactics and Hands-on Skills Sessions**
Introduction to the use of likelihood ratios and evidence based bedside medicine	Introduction to POCUS and use of an US machine (Knobology)
Clinical case: Pneumonia (with review of differential diagnosis and introduction to EBPE). Discussion of diagnostic utility of physical exam components in establishing a diagnosis of pneumonia and pleural effusions, with particular focus on egophony and percussion. Includes review of basic pulmonary auscultation, description of abnormal findings, and their clinical implications. Additional discussion on clinical predictors of pneumonia and radiographic findings as they relate to underlying infectious organism.	Lung: Protocolized approach to hypoxia assessment, normal and pathologic profiles.
Clinical case: Chest Pain (Acute Coronary Syndrome, Venous Thromboembolism)	DVT: Rule-in assessment using two-zone approach
Clinical cases: Syncope	Shock Assessment (IVC measurement, RUSH protocol, assessment for cardiac tamponade)
Clinical case: Congestive Heart Failure. Discussion of diagnostic utility of physical exam components in establishing heart failure, with particular focus on JVP assessment, PMI, predictors of valvular pathology based on murmur characteristics, and discussion of the Valsalva maneuver.	Focused Cardiac Ultrasound (FOCUS) using four views to assess for pericardial effusion, gross assessment of left ventricular function, and right ventricular size
Clinical Case: Cirrhotic Liver Disease	Abdominal US: RUQ, LUQ, Abdominal Aorta
Clinical Case: Gastrointestinal Bleeding	Procedures: Paracentesis, Thoracentesis, Joint Aspiration
Clinical Case: Abdominal Pain. Discussion of diagnostic utility of physical exam components (isolated maneuvers vs. diagnostic scores) in establishing a diagnosis of hepatomegaly, acute cholecystitis and acute appendicitis.	Abdominal US: Renal and Bladder
Clinical Case: Chronic Obstructive Pulmonary Disease	Scanning Workshop: Hands-on skills improvement and introduction to quality assessment and online free and open access medical education resources (FOAMed)
Clinical Case: Soft tissue pathology	Soft tissue and Musculoskeletal

**Table 2:** Primary and Secondary Resources for Course development.

**Primary Resources**	**Selected Secondary Resources**
**POCUS topics**	
Point-of-Care Ultrasound, Nilam J Soni, Robert Arntfield, Pierre Kory- Second Edition (2019; Elsevier)	Volpicelli G, Elbarbary M, Blaivas M, et al. International evidence-based recommendations for point-of-care lung ultrasound. Intensive Care Med. 2012;38(4):577–591. Via G, Hussain A, Wells M, et al. International evidence-based recommendations for focused cardiac ultrasound. J Am Soc Echocardiogr. 2014;27(7):683.e1–683.e33. Frankel HL, Kirkpatrick AW, Elbarbary M, et al. Guidelines for the Appropriate Use of Bedside General and Cardiac Ultrasonography in the Evaluation of Critically Ill Patients-Part I: General Ultrasonography. Crit Care Med. 2015;43(11):2479–2502. Levitov A, Frankel HL, Blaivas M, et al. Guidelines for the Appropriate Use of Bedside General and Cardiac Ultrasonography in the Evaluation of Critically Ill Patients-Part II: Cardiac Ultrasonography. Crit Care Med. 2016;44(6):1206–1227. Vandemergel X. Point-of-care ultrasound (POCUS) for hospitalists and general internists [published online ahead of print, 2019 Dec 9]. Acta Clin Belg. 2019;1–7. Fentress M, Heyne TF, Barron KR, Jayasekera N. Point-of-Care Ultrasound in Resource-Limited Settings: Common Applications. South Med J. 2018;111(7):424–433. Blanco P, Volpicelli G. Common pitfalls in point-of-care ultrasound: a practical guide for emergency and critical care physicians. Crit Ultrasound J. 2016;8(1):15.
**Evidence –Based Physical Exam topics**	
Evidence –Based Physical Diagnosis. Steven McGee. 4^th^ edition (2016; Elsevier) JAMAevidence. The Rational clinical examination; accessed via online subscription to JAMAevidence.com	Metlay JP, Fine MJ. Testing strategies in the initial management of patients with community-acquired pneumonia. Annals of Internal Medicine. 2003 Jan 21;138(2):109-18. Review. Fine MJ, et al. A prediction rule to identify low-risk patients with community-acquired pneumonia. New England Journal of Medicine. 1997 Jan 23;336(4):243-50. Albaum MN, et al. Interobserver reliability of the chest radiograph in community-acquired pneumonia. PORT Investigators. Chest 1996; 110:343. Bohadana A, et al. Fundamentals of lung auscultation. N Engl J Med. 2014 Feb 20;370(8):744-51. Schiavone W. Cardiac Tamponade: 12 pearls in diagnosis and management. CCJM. 2013;80(2):109-116 King M, et al. “Diagnosis and evaluation of heart failure.” Am Fam Physician. 2012 Jun 15;85(12):1161-8. Wang CS, et al. “Does this dyspneic patient in the emergency department have congestive heart failure?” JAMA. 2005 Oct 19;294(15):1944-56. Madhok V, et al. “The accuracy of symptoms, signs and diagnostic tests in the diagnosis of left ventricular dysfunction in primary care: a diagnostic accuracy systematic review.” BMC Fam Pract. 2008 Oct 8;9:56. doi: 10.1186/1471-2296-9-56. Felker GM, et al. “The Valsalva maneuver: a bedside "biomarker" for heart failure.” Am J Med. 2006 Feb;119(2):117-22. Barrett MJ, et al. Cardiac Auscultation in the Modern Era: Premature Requiem or Phoenix Rising? Cardiol Rev. 2017 Sep/Oct;25(5):205-210. Sibbald M, et al. Role of clinical context in residents’ physical examination diagnostic accuracy. Med Educ. 2011 Apr;45(4):415-21. Barrett MJ, et al. Mastering cardiac murmurs: the power of repetition. Chest. 2004 Aug;126(2):470-5. . Srygley FD, et al. Does This Patient Have a Severe Upper Gastrointestinal Bleed? JAMA. 2012;307(10):1072–1079.; Sharma SK, Aggarwal R. Prediction of large esophageal varices in patients with cirrhosis of the liver using clinical, laboratory and imaging parameters. J Gastroenterol Hepatol. 2007;22(11):1909–1915.

To overcome a common challenge of identifying patients with relevant pathology, our faculty developed reporting tools within our Electronic Medical Record (EMR- Epic) to identify patients with diagnoses relevant to the course. 

### Program’s Assessment

**Figure 1:**
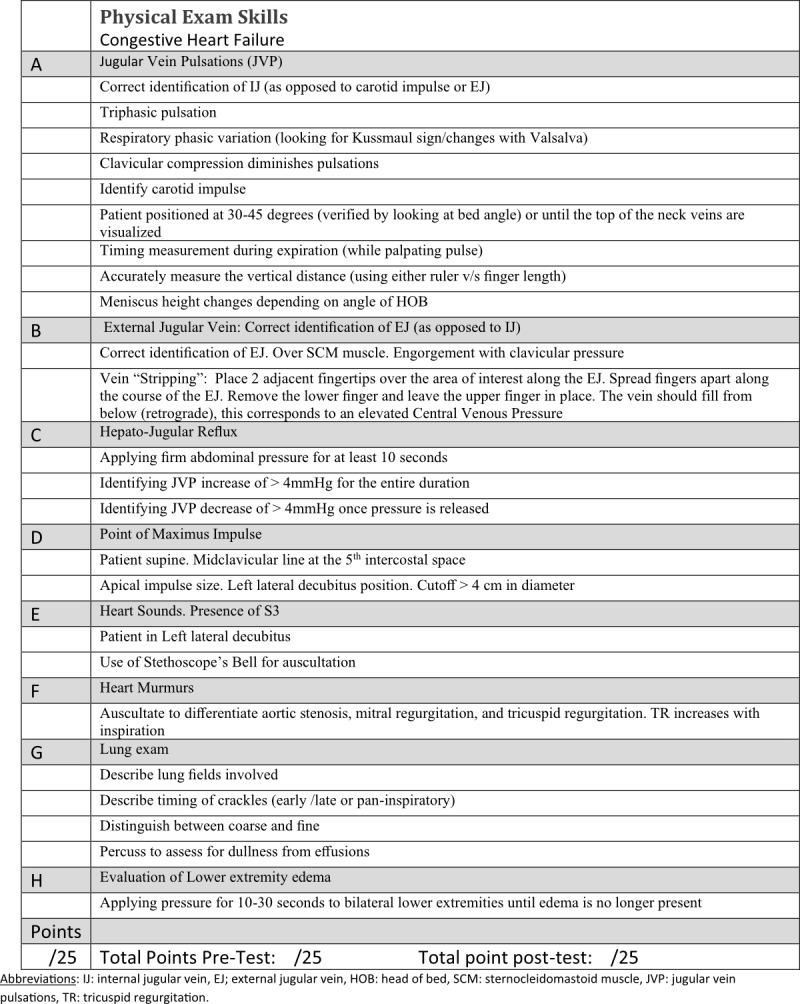
Checklist for Physical Exam Skills- Case: Congestive Heart Failure. Used for both Pre and Post Test.

We combined quantitative and qualitative measures. Beginning in 2016, all participants underwent a pre-test and post-test which involved direct observation of learners conducting the physical exam, followed by real-time feedback with the goal of motivating them to improve their physical examination skills. For the pre-test, participants were asked to perform a comprehensive cardiac examination for a patient with suspected heart failure, a comprehensive pulmonary exam for a patient with suspected pneumonia, a targeted exam for cirrhotic patients, and a focused neurologic examination. They were directly observed using a standardized checklist (Figure 1) to assess whether they correctly perform physical exam maneuvers recognized in the literature to have good predictive value for heart failure, pneumonia, and complications of cirrhosis.

## Results

**Figure 2:**
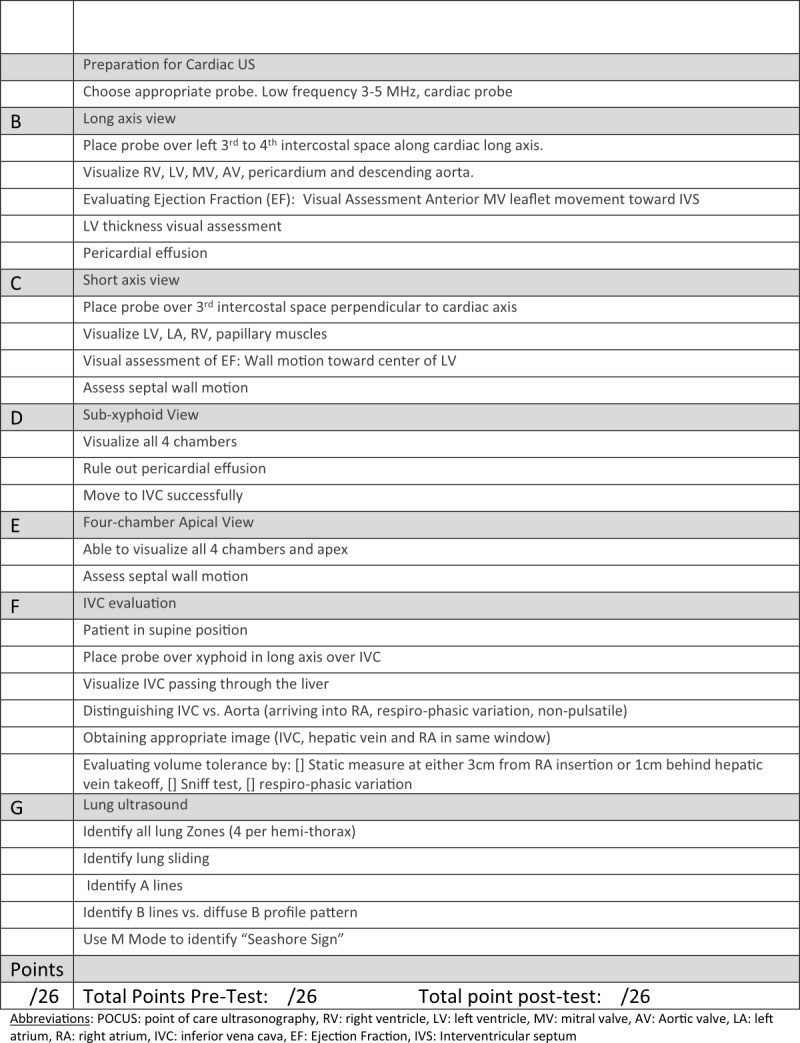
Checklist for Bedside Ultrasound Skills- Chest (heart and lung) POCUS. Used for both Pre and Post Test.

In 2019, the three participants that completed both pre- and post-tests (because of illness and scheduling conflicts, only 3 of 5 participants were observed pre and post)—correctly performed an average of 3.3 of 9 (range 0-7) components related to visual assessment of JVP in the pre-test and 4.7 in the post-post (range 4-6). Only 1 of 3 assessed the point of maximal impulse (PMI) in the pre-test; all 3 assessed PMI in the post-test. In the pre-test, no participant correctly assessed for egophony or asymmetric pulmonary expansion; all 3 assessed both in the post-test. Cirrhosis inspection and palpation scores improved from 0.67(range 0 -2) to 4.67 (range 3-6) (of 6) and 2 (range 1-3) to 4 (range 3-5) (of 5) pre- to post-test, respectively (Table 3). A twenty-six point checklist created by the POCUS faculty was used to perform a pre-elective hands-on cardiac and lung POCUS skills assessment. The US skill checklist is shown in Figure 2. This assessment tested knowledge and performance of ultrasound probe and machine functions, relevant anatomy focused on standard views of the heart and lungs, and basic diagnostic POCUS assessments of these organs. The median pre-test score was 9 out of 26 possible points. 

**Table 3:** EBPE Pre- and Post-test Mean Scores

**Exam Maneuver** ^*^	**Average Pre-test score**	**Average Post-test score**
Egophony (out of 1)	0 [0-0]	1 [1-1]
Asymmetric expansion (out of 1)	0 [0-0]	1 [1-1]
Cirrhosis inspection (out of 6)	0.67 [0-2]	4.67 [3-6]
Cirrhosis palpation (out of 5)	2 [1-3]	4 [3-5]
Cirrhosis maneuvers (out of 2)	1 [1-1]	1 [1-1]
Neuro exam (out of 11)	3.67 [3-5]	2.67 [2-3]

At the conclusion of the elective, participants prepared and delivered small group teaching sessions to junior learners, discussing the evidence-based elements of the cardiac and pulmonary examinations. They also demonstrated and led learners in the proper examination techniques on actual patients. Their teaching was directly observed, and they received feedback on their teaching techniques. All participants were effectively able to describe the pathophysiology underlying abnormal exam findings, demonstrate the highest yield physical exam maneuvers, and explain the method of acquisition and clinical implications of basic cardiac and pulmonary POCUS. At the end of the elective the POCUS hands-on skills assessment was repeated with an improvement of the median to 26 of 26 points. 

We also included components not covered in the course (neurologic examination) to serve as a control measure for our pre- and post-test exams. The average score on the neurologic pre-assessment was 3.67 (range 3-5) and the post course assessment average score was 2.67 (range 2-3).

The Master Clinician Elective underwent continuous comprehensive evaluation following the Plan-Do-Check-Act (PDCA) format, widely known as a strategic planning modality. Learners were asked to provide constructive feedback on rotation structure, individual didactic sessions, and faculty at course end. 

## Discussion

In the era of high value care, we believe it imperative for all future practitioners to master EBPE and POCUS skills. Because faculty and ultrasound resources are limited, we are working to expand our ability to teach this important content through both internal faculty development and partnering with other IMRP clinical sites. Our data clearly shows a marked improvement of learners in both their physical exam and POCUS skills.

While our elective is a collaboration of four physical exam-focused providers and two POCUS providers, it could feasibly be run with as few as 1 of each with enrollment limited to four participants. One of the challenges for our faculty and for faculty at other programs is limited protected time for POCUS education. Our study demonstrates the value of this elective, which should support decisions regarding allocation of protected time for faculty to dedicate to POCUS education. 

This elective requires the availability of bedside ultrasound technology and faculty with a level of expertise to accurately teach bedside ultrasound applications. In our experience, learners require approximately 1 machine for every 2 to 3 learners to have adequate scanning time to achieve course ultrasound goals. Our POCUS expert faculty collaborated in the curriculum design and reflected their experience as hands-on educators at a national level. They are invited speakers and faculty at annual conferences for the American College of Physicians, Society of Hospital Medicine (SHM), American College of Chest Physicians (CHEST), and Society of General Internal Medicine. A low number of learners per machine is a design tool used at the national level to maintain learner engagement and maximize individual teachable moments for this hand-eye skill. The incorporation of unguided scanning time with post-image acquisition quality review mimics on a small-scale the portfolio building processes used by both CHEST and SHM certification programs.

Our study is single-center and, by design, small in size. We believe it would be easily reproduced at other institutions, with limitations. The limitations are, in order of anticipated impact, a requirement of faculty skilled and capable of teaching POCUS and assessing skill and image acquisition of novice learners, access to ultrasound devices, faculty capable and skilled in teaching EBPE. While there are standards for what constitutes quality image acquisition and interpretation, there is inherent subjectivity in a bedside assessment of the learner. Furthermore, the assessment of learners was not blinded and was conducted by course faculty, leading to a possible bias of skills assessment and is a limitation of our findings.

Other considerations include the fact that we did not assess skill decay. The optimal frequency of “refresher” courses that would allow knowledge and skill retention is yet to be determined. 

We recognize that many GME programs offer POCUS training 8, 9, 10. The University of Toronto has published the only other curriculum available specifically combining physical exam and POCUS, but with only subjective evaluations available 11. We believe our elective is novel in its integration of EBPE and POCUS and its focus on objectively demonstrating skills acquisition that can impact the provision of timely, high value care for patients at a safety net hospital. This educational model allows us to utilize our limited resources effectively, rekindles enthusiasm for using the physical exam, improves physical exam skills and POCUS among medical trainees, and fosters their interest and ability to teach these important tools.

## Conclusion

An elective designed specifically to integrate POCUS and physical exam modalities improves the ability of resident physicians to utilize both diagnostic modalities. This elective enhances clinical reasoning by weaving traditional EBPE with novel POCUS, however little is known regarding the clinical impact of this training paradigm. How would this change medical imaging ordering practices? Will the positive yield of these orders increase? Will this process decrease or increase length of stay and overall hospital cost? These are amongst some of our high priority questions.

## Conflicts of Interest

None declared.
